# Glucocorticoid impact therapy for recurrent IgG4-related disease with diabetes insipidus as the main manifestation: A case report and literature review

**DOI:** 10.1097/MD.0000000000036129

**Published:** 2023-11-17

**Authors:** Yongzhuo Yu, Lili Xu, Yunyang Wang, Wenxuan Li, Yangang Wang

**Affiliations:** a Department of Endocrinology, The Affiliated Hospital of Qingdao University, Qingdao, China.

**Keywords:** case report, central diabetes insipidus, IgG4-related disease, IgG4-related hypophysitis, lacrimal gland enlargement

## Abstract

**Rationale:**

There is a relative wealth of experience in the initial treatment of IgG4-related disease (IgG4-RD), but little is known about therapeutic measures for recurrent cases combined with multiple organ and tissue involvement.

**Patient concerns:**

A 43-year-old man with a previous diagnosis of IgG4-RD due to recurrent right lacrimal gland enlargement with eyelid erythema presented with diabetes insipidus

**Diagnoses:**

We performed a pituitary Magnetic Resonance Imaging which revealed posterior pituitary rim changes with inhomogeneous enhancement and nodular-like thickening of the pituitary stalk, and performed a water-deprivation-vasopressin test confirmed central diabetes insipidus, and in combination with the patient’s elevated IgG4 levels and past medical conditions, we diagnosed central diabetes insipidus, IgG4-related hypophysitis, and IgG4-RD.

**Interventions:**

After the patient was admitted to the hospital we gave methylprednisolone 500 mg intravenously once daily for 4 days and again for 4 consecutive days after a 10-day interval. During this period combined with mycophenolate mofetil 250 mg twice daily and desmopressin acetate 0.1 mg 3 times daily.

**Outcomes:**

The patient was followed up for a sustained period of 6 months and no side effects of glucocorticoid therapy were noted, there were no signs of recurrence, and the daily urine output stabilized in the normal range.

**Lessons:**

We recognized that IgG4 levels do not reflect relapse or long-term control, and that glucocorticoid shock therapy is an optional and reliable treatment strategy for relapsed patients.

## 1. Introduction

IgG4-related hypophysitis (IgG4-RH) is a manifestation of IgG4-related disease (IgG4-RD) involving the pituitary gland and is a relatively rare form of pituitary inflammation of varying severity, often with involvement of other organs. We report the clinical data of a patient with IgG4-related pituitary inflammation admitted to our hospital in November 2022, who was seen for diabetes insipidus and had a relapse of IgG4-RD, and whose symptoms improved after glucocorticoid impact therapy. There is a wealth of research on initial treatment, but the choice of medication for patients with relapses is still poorly understood. Based on this case, we reviewed and summarized the relevant literature to improve clinicians’ understanding of this disease.

## 2. Case presentation

### 2.1. General information

**Figure 1.**
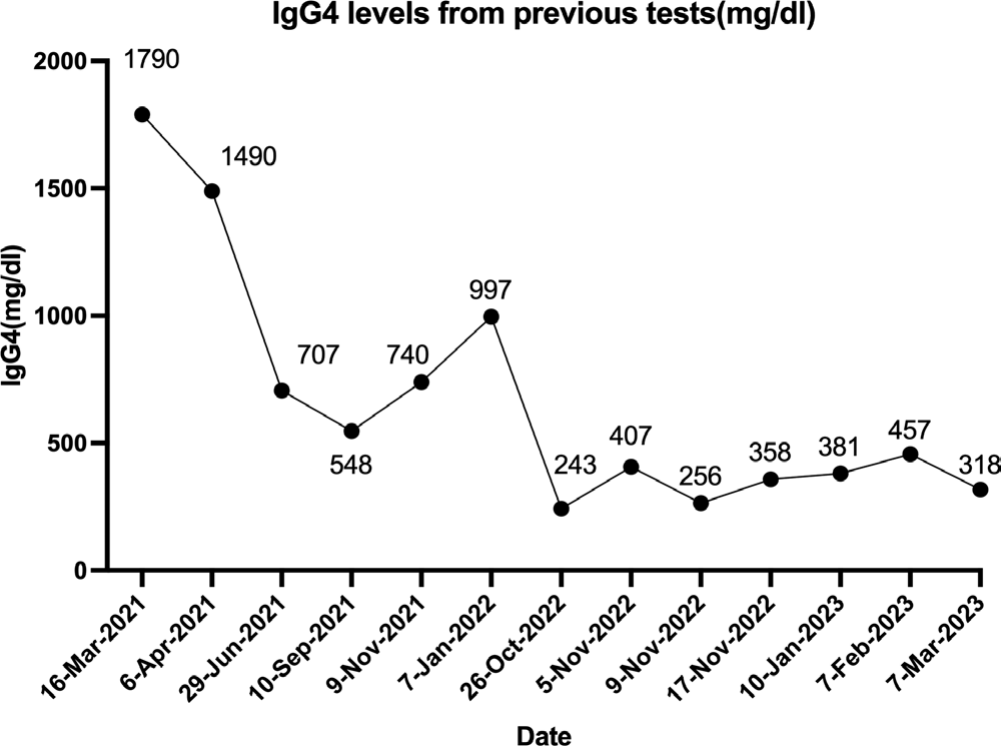
IgG4 levels from previous tests (mg/dL).

The patient, a 43-year-old male, had recurrent right lacrimal gland enlargement with eyelid erythema in early 2019 with no obvious cause, and his symptoms did not improve significantly after several visits to our ophthalmology department for treatment. Axial CT scan of the orbit suggested slight swelling of the right upper eyelid, bone changes in the right infraorbital wall and local soft tissue density. Further refinement of the immunoglobulin IgG4 assay 1790 mg/dL (reference range 3–201 mg/dL, see Fig. 1 for historical serum IgG4 levels) was diagnosed as IgG4-RD in April 2021. The patient was given methylprednisolone 12 mg once daily and mycophenolate mofetil (MMF) 0.25 g twice daily. During this period, the patient took the medication regularly and his symptoms were well controlled with no recurrence of eye discomfort.

**Figure 2.**
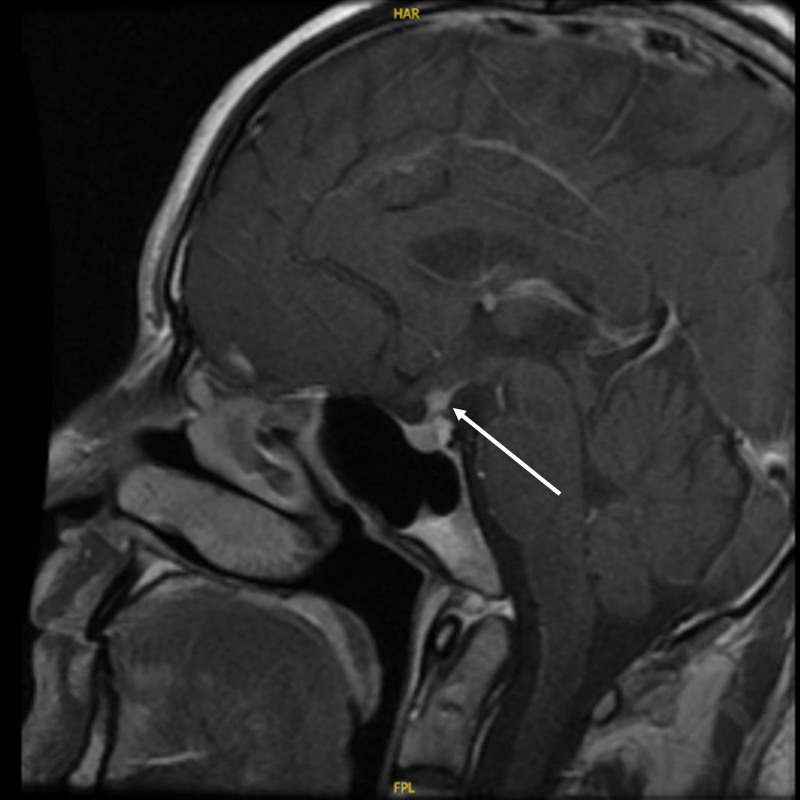
Pituitary MR dynamic enhancement films before impact therapy: posterior pituitary rim changes with inhomogeneous enhancement and nodule-like thickening of the pituitary stalk are seen (sagittal position, marked by arrows).

**Figure 3.**
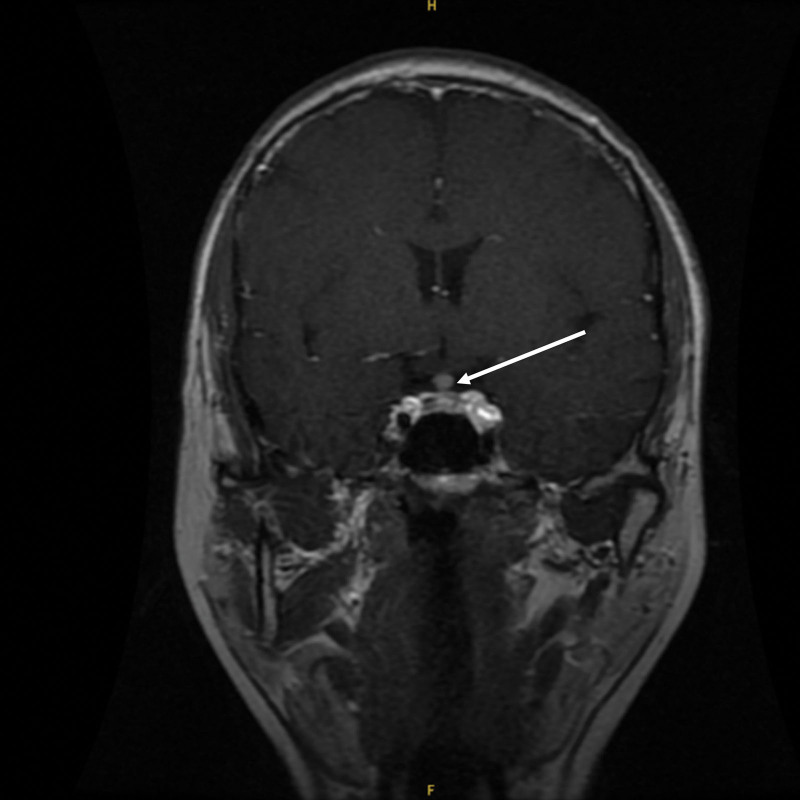
Pituitary MR dynamic enhancement films before impact therapy: posterior pituitary rim changes with inhomogeneous enhancement and nodule-like thickening of the pituitary stalk are seen (coronary position, marked by arrows).

In September 2022, the patient developed polyhydramnios and polyuria with strong thirst without any obvious trigger, drinking more than 4000 mL of water daily with similar volume of clear urine. The patient then visited our endocrinology outpatient clinic on October 25, 2022, and was tested for immunoglobulin G4 243 mg/dL, and pituitary MR dynamic enhancement examination suggested posterior pituitary rim changes with inhomogeneous enhancement and nodular-like thickening of the pituitary stalk (Figs. 2 and 3). The patient was diagnosed with central diabetes insipidus (CDI), IgG4-RH, and given desmopressin tablets 0.1 mg twice daily, the patient’s urine output decreased compared to before, but remained between 2500 and 3500 mL. The symptoms did not improve significantly, so he was admitted to our endocrinology department on November 4, 2022.

The patient had a previous history of hypertension for more than 10 years with a maximum blood pressure of 150/90 mm Hg, which was controlled with Irbesartan 150 mg once daily and could be controlled within the normal range in the usual way. On admission, blood pressure was 149/87 mm Hg, skin was dry, and the rest had no specific positive signs, suggesting fair volume and no significant dehydration.

### 2.2. Laboratory and imaging test

The patient was discontinued from desmopressin tablets upon admission, and a water-deprivation-vasopressin test was performed the following day. The patient’s urine osmolality was 243.50 mOsm/L at night the previous day and 171.4 mOsm/L after overnight water abstinence, at which time the patient showed signs of hypovolemia and lower blood pressure than before; 5 U of vasopressin was given subcutaneously, and the urine osmolality was subsequently measured at 313.0 mOsm/L (∆urinary osmolality 82.6%), which was identified as CDI.

We also examined the other hormonal axes associated with the pituitary gland. For thyroid-related, anti-thyroglobulin antibody 11.80 IU/mL (reference range 0–15.00 IU/mL), thyroid-stimulating hormone 4.19 uIU/mL (0.27–4.20 uIU/mL), free triiodothyronine 5.07 pmol/L (3.10–6.80 pmol/L), free thyroxine 14.50 pmol/L (12.00–22.00 pmol/L), and anti-thyroid peroxidase antibody <9.00 IU/mL (reference range 0–34.00 IU/mL). Sex hormone related, prolactin 793.60 mIU/L↑ (86.00–324.00 mIU/mL), estradiol 122.20 pmol/L (99.40–192.00 pmol/L), luteinizing hormone 7.85 mIU/mL (1.70–8.60 mIU/mL), follicle stimulating hormone 12.30 mIU/mL (1.50–12.40 mIU/mL), progesterone 0.38 nmol/L (0.16–0.47 nmol/L), and testosterone 17.35nmol/L (9.90–27.80 nmol/L). Growth hormone 0.28 ng/mL (0.03–2.47 ng/mL), insulin-like growth factor 73.60 ng/mL (60–350 ng/mL), (8 am) adrenocorticotropic hormone 21.50 pg/mL (7.20–63.3pg/mL), and (8 am) cortisol 185.00 nmol/L (166–507 nmol/L).

At the same time, we performed CT examination of the chest and ultrasonography of the thyroid, liver, gallbladder, pancreas, spleen and both kidneys, and no other organ involvement was found. In addition, complete blood count, urine routine, stool routine and occult blood, blood coagulation routine, liver function, kidney function, blood calcium, blood phosphorus, blood magnesium, uric acid did not show any significant abnormalities. Tumor markers, neuron-specific enolase, CA19-9, methemoglobin, and specific beta human chorionic gonadotropin were also within the normal range.

### 2.3. Treatment and follow-up

Combined with the history, physical examination and ancillary test results, the diagnosis was considered (1) CDI; (2) IgG4-RD, IgG4-RH; and (3) hypertension grade 1 (low risk). Methylprednisolone 500 mg IV once daily was given on November 4 to 8 and November 17 to 20, 2022, respectively. Combined with MMF 250 mg twice daily and desmopressin acetate 0.1 mg 3 times daily. The patient’s elevated prolactin was considered a pituitary stalk effect and bromocriptine 1.25 mg was given once a night. During the first phase of impact therapy, the patient’s thirst symptoms improved, and urine output gradually changed to 1500 to 2000 mL/day.

**Figure 4.**
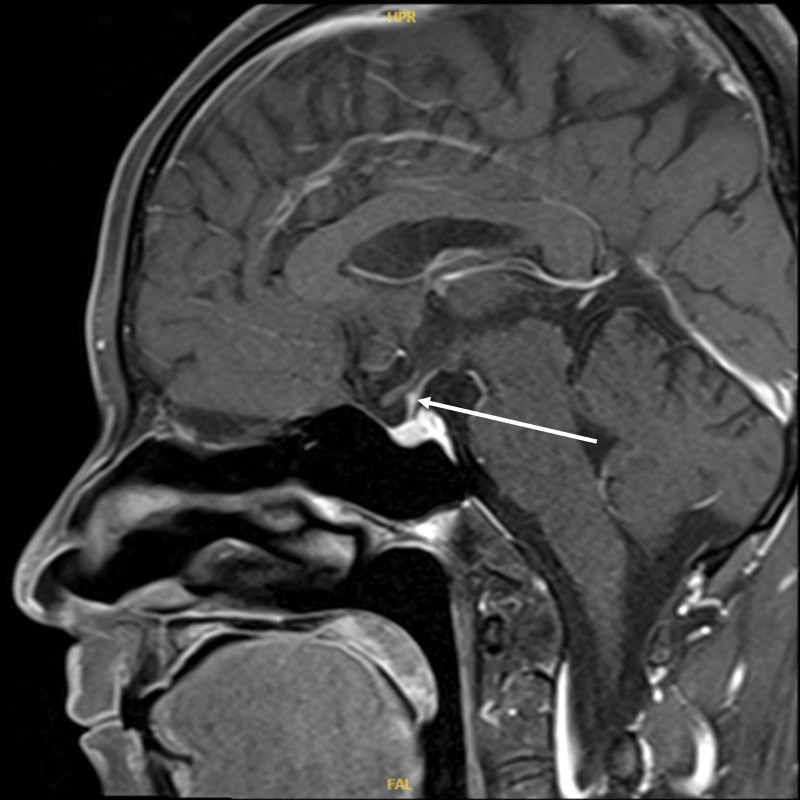
Post-treatment pituitary MR dynamic enhancement films: pituitary stalk nodes are significantly smaller than before (sagittal position, marked by arrows).

**Figure 5.**
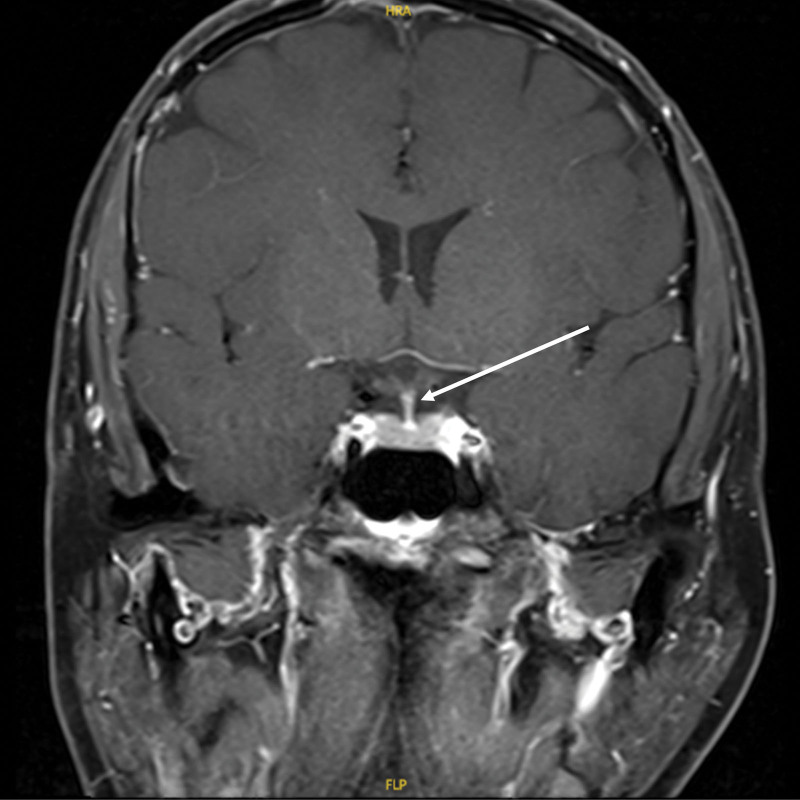
Post-treatment pituitary MR dynamic enhancement films: pituitary stalk nodes are significantly smaller than before (coronary position, marked by arrows).

A repeat pituitary MR enhancement scan on November 21, 2022, suggested that the pituitary stalk nodes were significantly smaller than before (Figs. 4 and 5). After discharge, the patient’s glucocorticosteroids were tapered and he is currently on methylprednisolone 4 mg once daily and MMF 250 mg twice daily. By June 2023, patients were followed up regularly, no side effects of glucocorticoid therapy were observed, no signs of relapse, and the daily urine output was stable at 1500 to 2000 mL. In addition, the patient’s prolactin returned to normal during the follow-up period, whereas IgG4 was consistently higher than normal in patients as in Figure 1.

## 3. Literature review

We used “hypophysitis,” “IgG4-related hypophysitis,” “diabetes insipidus” as keywords and searched on PubMed database. The review literature has been available for 115 cases of IgG4-RH before 2021,^[1]^ so the search period was from January 1, 2021 to December 1, 2022, and 15 case reports were detected,^[2–16]^ including 12 with complete clinical information and a total of 24 patients^[2–7,9–14]^ (Table 1).

**Table 1:** Characteristics of cases of IgG4-associated pituitary inflammation (in chronological order of reporting).

No.	Reporters (first author)	Gender	Age (yr)	Age of onset of IgG4-RD (or IgG4-RH) (yr)	IgG4 (mg/dL)	Pathological examination	Hypopituitarism	CDI	Involvement of other organs	Glucocorticoids	Drug combination
1^[2]^	Arya	F	11	11	19	Pituitary	–	–	–	+	MMF
2^[3]^	Gersey	F	63	63	57	Pituitary	+	+	–	+	Rituximab
3^[4]^*	Kimura	M	12	12	1093	NA	–	+	–	–	–
4^[5]^	Meng	M	61	53	4370	Submandibular gland	NA	+	Lacrimal gland, submandibular gland	+	CTX
5^[5]^	Meng	M	60	58	225	NA	NA	–	Pancreas	+	CTX
6^[5]^	Meng	M	57	56	341	Lymph nodes	NA	+	Pancreas, lung, pleura, lymph nodes, parotid gland, submandibular gland, bile duct	+	CTX, Rituximab
7^[5]^	Meng	F	56	44	1880	Lacrimal gland	NA	+	Pancreas, lung, kidney, lacrimal gland, submandibular gland, parotid gland	+	CTX, MMF, Rituximab
8^[5]^	Meng	M	63	40	2510	Pancreas	NA	+	Pancreas, kidneys, ureters, lymph nodes	+	Azathioprine
9^[5]^	Meng	M	50	43	3270	Lymph nodes	NA	+	Lacrimal gland, lymph nodes	+	–
10^[6]^	Nishi	M	71	71	264	Submandibular gland	+	+	Submandibular gland	+	–
11^[7]^	Urushida	M	79	79	157	NA	–	+	–	+	–
12^[9]^	Yoshida	F	43	43	462	Right orbital mass	+	+	Meninges, right orbital	+	–
13^[10]^†	Bhargava	F	44	44	NA	Pituitary	+	-	NA	-	–
14^[10]^†	Bhargava	M	26	26	51	Pituitary	+	+	NA	-	–
15^[10]^†	Bhargava	M	60	60	NA	Pituitary	+	+	NA	-	–
16^[10]^†	Bhargava	M	74	74	500	Pituitary	+	–	NA	+	–
17^[10]^†	Bhargava	M	36	36	NA	Pituitary	+	–	NA	–	–
18^[10]^†	Bhargava	M	58	58	108	Pituitary	+	–	NA	–	–
19^[10]^†	Bhargava	F	62	62	NA	Pituitary	+	+	NA	+	–
20^[10]^†	Bhargava	F	44	44	197	Pituitary	+	–	NA	+	–
21^[11]^	Haj Mohamad Ebrahim	F	33	33	141	Right orbital mass	–	–	Right orbital	+	Azathioprine
22^[12]^	Huang	M	53	52	NA	Liver	–	–	Bile ducts, pancreas, salivary glands, lacrimal glands	+	–
23^[13]^	Iwamoto	M	45	44	792	Labial gland	–	+	Submandibular gland, parotid gland, cervical lymph nodes	+	–
24^[14]^	Lv	M	47	47	298	Pituitary	+	–	–	+	–

Note: “+” = the symptom is present or the drug is used; “–” = the symptom is not present or the drug is not used; “NA” = the test is not mentioned in the report or not performed.

CDI = central diabetes insipidus, CTX = cyclophosphamide, F = female, IgG4-RD = IgG4-related disease, IgG4-RH = IgG4-related hypophysitis, M = male, MMF = mycophenolate mofetil.

* This patient was administered desmopressin only and no anterior pituitary dysfunction or progressively enlarged pituitary lesions were observed during the subsequent 2-year follow-up period, and the patient grew normally.

† The patients are from the same case report and all underwent pituitary surgery.

“Complete clinical information” is defined as including complete information on sex, age, time of onset, symptoms, laboratory tests, pituitary function tests, imaging data, treatment and follow-up prognosis, and a diagnosis in accordance with the 2020 Japanese revised IgG4-RD comprehensive diagnostic criteria and/or the 2019 American College of Rheumatology (ACR)/European League Against Rheumatism (EULAR) international classification criteria,^[17,18]^ and the diagnostic criteria for IgG4-RH proposed by Leporati et al.^[19]^ Some cases reporting no IgG4 levels or biopsy pathology but meeting the above diagnostic criteria were also included.

Among them, Amirbaigloo et al^[1]^ reviewed 115 cases of IgG4-RH up to 2021 is the largest number of cases included so far, found that IgG4-RH was predominantly male (72, 62.6%) and predominantly middle-aged and elderly (median 56 years, range 14–87 years). Diabetes insipidus was one of the common symptoms, and in the 115 cases with posterior pituitary function described in 89 cases, 70 patients had diabetes insipidus (78.7%). Anterior pituitary function was described in 102 cases, of which 86 patients had varying degrees of hypopituitarism and various hormone deficiencies (84.3%). In addition, 87 of them reported specific serum IgG4 levels with a mean of 666.2 ± 936.4 mg/dL.

## 4. Discussion

CDI refers to varying degrees of polyuria due to reduced release of antidiuretic hormone from various causes, caused by hypotensive or disrupted neuronal function in the supraoptic and paraventricular nuclei of the hypothalamus. The most common cause is idiopathic diabetes insipidus (30%–50%),^[20,21]^ while others include primary or secondary tumors, infections, cranial surgical trauma,^[22]^ trauma,^[23]^ autoimmune and, less commonly, genetic factors.^[24,25]^ In this patient, CDI and lacrimal gland enlargement were the main clinical features, and the serum IgG4 level was significantly elevated, combined with imaging features and good response to glucocorticoid therapy, so the final diagnosis of IgG4-RH was made. It suggests that in clinical practice, the possibility of systemic diseases including IgG4-RD should be considered in CDI with multiple site or organ involvement.

IgG4-RD is a class of fibroinflammatory diseases in which involvement of almost every organ has been reported.^[26,27]^ the exact incidence and prevalence of IgG4-RD is unknown, and current statistics suggest that the disease is slightly more prevalent in middle-aged and older men,^[28]^ and a prospective study conducted in China found a similar gender composition.^[29]^ Involved organs also vary by gender, for example, renal and retroperitoneal lesions are more commonly seen in males, while salpingitis, lacrimation and primary biliary cirrhosis are more prevalent in females.^[29]^ For the patient in this case, we also conducted some further examinations and found no involvement of other organs.

**Figure 6.**
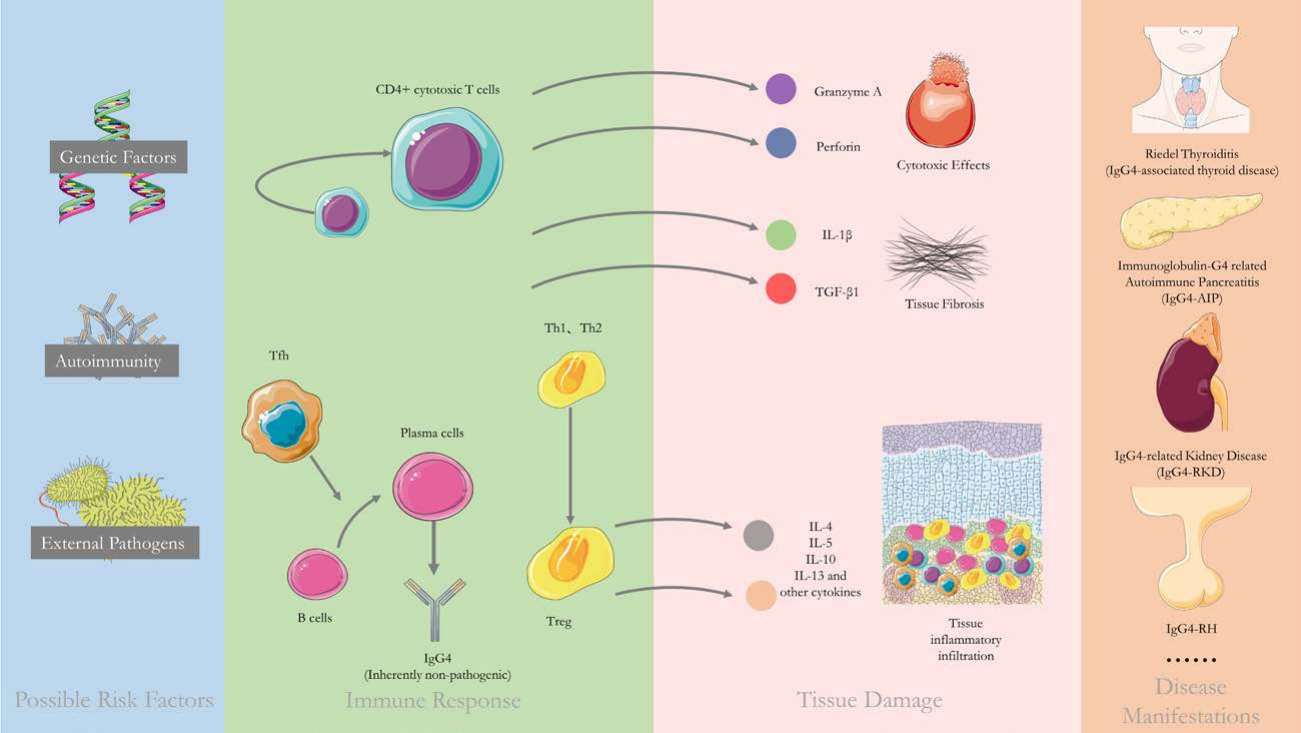
Pathogenesis of IgG4-RD. It is currently believed that genetic factors, bacterial infection, and autoimmunity act simultaneously to cause the disease, and that clonally proliferating CD4+ cytotoxic T cells may be the key to pathogenesis, secreting granzyme A and perforin to exert cytotoxic effects, while releasing cytokines such as IL-1β and TGF-β1 to cause fibrosis.

For the pathogenesis of IgG4-RH, or more generally IgG4-RD, it is currently, believed that there is a role for both genetic risk factors, bacterial infection, and autoimmunity,^[27]^ but the exact etiology is unclear. Mattoo et al^[30]^ found clonally proliferating CD4+ cytotoxic T cells in both the peripheral blood and the lesions of the patients, and they hypothesized that these cells were key to the pathogenesis. (Fig. 6) The patient’s IgG4 level was always higher than normal during the course of the disease, and the patient’s IgG4 level at the time of new onset of CDI was the lowest ever detected, probably due to the relatively long life span of plasma cells, which can secrete IgG4 for a long time. Similarly, we can find that IgG4 levels do not predict or reflect disease control or relapse. Further studies also found that increased IgG4 antibodies may have an anti-inflammatory tendency and that specific anti-IgG4 autoantibodies have not been found in patients,^[27]^ suggesting that IgG4 antibodies themselves are not pathogenic and that their overexpression may be the response to primary inflammatory stimuli.

The fact that no biopsy was performed during the treatment of this patient is a regret of the whole treatment process, but combined with the patient’s previous history of IgG4-related ophthalmopathy and the available tests, we can exclude CDI caused by primary or secondary tumors, infections, cranial surgical trauma, etc., and the diagnosis of IgG4-RH is clear in accordance with the diagnostic criteria for IgG4-RH proposed by Leporati et al^[19]^ mentioned above. It should also be emphasized that pathological changes are an important basis for the diagnosis of IgG4-RD. The typical pathology is a dense lymphocytic tissue infiltration with storiform pattern,^[31]^ which may be accompanied by obliterative phlebitis and mild to moderate eosinophilic infiltration, and this pathological change is similar between the different organs involved.^[27]^

In terms of treatment, IgG4-RH also follows the IgG4-RD approach, with guidelines recommending glucocorticoids as the first-line treatment in the presence of symptomatic activity. The natural course and prognosis of IgG4-RD are not fully understood, but there is a clear tendency for IgG4-RD to recur,^[32]^ and most of the original case reports were for cases with initial onset. We emphasize that recurrence of IgG4-RD may involve more organs and tissues, and in this case, the patient had a new onset of pituitary inflammation and CDI based on long-term control of the primary symptoms. There are not many references for the treatment of relapsed patients. Of the 24 patients we summarized, 3 patients who reported recurrence during follow-up, but the description of treatment for the relapses was less clear in all cases. In this case, the patient’s symptoms improved significantly after methylprednisolone impact therapy combined with MMF, suggesting that glucocorticoids are still effective in relapsed patients and that impact therapy is an optional dosing modality.

In conjunction with the above, we recommend that in patients with relapses and new organ involvement, methylprednisolone impact therapy can be considered at a dose of 500 to 1000 mg per day, with a lower dose chosen when combined with immunosuppressive drugs for 3 days, followed by a 10% to 20% reduction every 1 to 2 weeks, gradually decreasing to a maintenance dose that prevents relapse. In addition, some patients were not treated with glucocorticoids during their previous therapy and can be discontinued directly after the effect of shock therapy within 2 weeks, which is unlikely to trigger suppression of the hypothalamic-pituitary-adrenocortical axis.^[33]^ In the initial treatment, drug combinations have been shown to provide greater benefit, and a similar therapeutic effect may have been seen in this case of relapse. In addition, considering the tendency of IgG4-RD to recur, patients need to be followed up regularly over a long period of time, and they also need to be able to report new symptoms in a timely manner.

## 5. Conclusion

Overall, IgG4-RD is a class of fibrous inflammatory diseases that can involve multiple organs throughout the body, and the involvement of different organs can occur sequentially. Pituitary gland involvement can result in CDI, anterior hypopituitarism or total hypopituitarism, and glucocorticoids are currently the recommended effective treatment. Although the number of reported cases of IgG4-RH has gradually increased since the first case in 2004,^[34]^ the disease has not been recognized for a long time. Combined with the literature review above, the epidemiology, pathogenesis and treatment of IgG4-RH need to be further improved, and there are not many references for the treatment of relapsed patients in clinical practice. We recognize from the treatment of this patient that IgG4 levels do not reflect relapse or long-term control, and that glucocorticoid shock therapy is an optional and reliable treatment strategy for relapsed patients, the treatment process of this case is expected to enrich the knowledge of disease treatment.

## Author contributions

**Conceptualization:** Yongzhuo Yu.

**Supervision:** Yangang Wang.

**Writing – original draft:** Yongzhuo Yu.

**Writing – review & editing:** Lili Xu, Yunyang Wang, Wenxuan Li.
